# News and Notes

**Published:** 1908-02

**Authors:** 


### NEWS AND NOTES

The Tri-State Medical Association of the Carolinas and Virginia will hold their next annual meeting in Charlotte on Feb. 18th and 19th.

The thirty-ninth regular semi-annual meeting of the Southern California Medical Society was held at Riverside, Cal., December 4th, 5th, 1907, at the Hotel Glenwood.

Dr. C. S. Harle, Chihuahua, sentenced to death for the murder of two Americans in order to collect their life insurance, has been commuted to imprisonment for twenty years.

It is with regret that we note the death of one of our oldest subscribers, Dr. W. B. Barton, who died of pneumonia at his home in Bartow, Ga.

The Nobel prize for the present year "for the greatest benefaction to mankind by a discovery in medicine in recent years" was conferred on Alphonse Laveran, the discoverer of the germ of malaria.

The University of Heidelberg recently conferred the Moos prize on Marie Kobele, a medical student from Baden. The prize amounted to $225. It is stated to be the first time in Germany that an academic prize has been conferred on a female medical student.

---

King Edward has bestowed the Order of Merit on Miss Florence Nightengale. The recipients of this order, which was instituted by him as a recognition of distinguished services to art or science, number only twenty, and Miss Nightengale is the only woman on whom it has been conferred.

---

On Nov. 4th, an ordinance was passed by the City Council, of Chicago, establishing "Zones of Quiet," which concerns the territory within 250 feet of each hospital. No disturbance or unnecessary noise is permitted within this area and anyone violating such laws will be subject to fine of from $2.00 to $50.00.

---

The students of the University of Pennsylvania have formed an Under-graduates Medical Association, similar to the American Medical Association, for the purpose of encouraging original research and a scientific spirit. The organization already has over two hundred and fifty members.

---

At a recent meeting of the Chattahoochee Valley Medical Association, in Columbus, Ga., the following officers were elected for the ensuing year: President, J. H. McDuffie of Columbus; vice-president, J. G. Palmer of Opelika; second vice-president, James J. Wynn of Clayton; secretary, W. J. Love of Opelika; treasurer, A. J. Coley of Alexander City. T. E. Mitchell of Columbus, and J. H. Drake of Auburn, were elected members of the board of council.

---

The partnership recently formed between Dr. A. W. Stirling and Dr. Frank M. Cunningham has been dissolved.

Dr. Cunningham will not return to Atlanta, and Dr. Stirling will continue his practice as formerly.

The New York Legislature has passed an act making it a felony to sell cocaine except on a written prescription.

---

Dr. Kenneth W. Millican, of Chicago, was tendered a banquet at the Columbian Club, Saint Louis, Friday evening, November 8, 1907, which was attended by sixty of his professional friends. A number of excellent speeches were made and a gold watch was presented to Dr. Millican in token of the esteem in which he was held by the Saint Louis profession.

---

Beginning with January *The American Journal of Urology* will be edited by Dr. William J. Robinson, editor of the *Critic and Guide, Therapeutic Medicine,* etc. The journal will be enlarged in scope so as to include venereal and skin diseases and there will be added an abstract department which will review the genito-urinary and dermatologic literature in every civilized language. The subscription price has been reduced to $2.00. The publication and editorial offices have been removed to 12 Mt. Morris Park West, New York City.

